# Ferroptosis in Radiation-Induced Ovarian Injury: Molecular Mechanisms and Emerging Therapeutic Strategies

**DOI:** 10.3390/antiox15101298

**Published:** 2026-10-07

**Authors:** Mody Albalawi, Sahar Khateeb

**Affiliations:** 1 Department of Biochemistry, Faculty of Science, University of Tabuk, Tabuk 71491, Saudi Arabia; 2 Biochemistry Division, Department of Chemistry, Faculty of Science, Fayoum University, Fayoum 63514, Egypt

**Keywords:** radiation, ovarian injury, ferroptosis, GPX4, lipid peroxidation, iron homeostasis, female fertility

## Abstract

Radiation therapy is one of the most common treatments for cancer. However, side effects on normal tissues remain a major clinical concern. The ovary is highly radiosensitive, and ionizing radiation (IR) can impair ovarian function and fertility by inducing oxidative stress, mitochondrial dysfunction, and multiple forms of cell death, ultimately leading to follicular depletion. Ferroptosis is an iron-dependent form of regulated cell death characterized by lipid peroxidation and has recently emerged as a potential contributor to IR-induced ovarian injury. IR can induce ferroptosis by disrupting iron homeostasis, impairing the System Xc^−^–GSH–GPX4 antioxidant defense pathway, and promoting lipid peroxidation. Recent preclinical studies have shown ferroptosis-related alterations in irradiated ovarian tissue and granulosa cells. In parallel, IR activates the well-established TAp63–PUMA/NOXA apoptotic pathway in oocytes, highlighting the involvement of multiple cell death mechanisms in ovarian injury. Nevertheless, targeting ferroptosis may help reduce granulosa cell injury and mitigate certain aspects of ovarian damage. This review evaluates current evidence for ferroptosis as one of the cell death mechanisms contributing to IR-induced ovarian injury, with particular emphasis on its molecular mechanisms, interactions with other cell death pathways, and emerging therapeutic strategies. Potential therapeutic strategies include the preservation of GPX4-dependent antioxidant defenses, inhibition of lipid peroxidation, regulation of iron homeostasis, and modulation of polyunsaturated fatty acid-related lipid metabolism. Nevertheless, direct evidence remains limited, and further research is needed to clarify the contribution of ferroptosis to IR-induced ovarian injury and evaluate whether targeting this pathway can mitigate ovarian damage without compromising tumor radiosensitivity.

## 1. Introduction

Ionizing radiation (IR) is used as a cytotoxic therapy [1], with around 50% of cancer patients undergoing radiation treatment [2,3]. Despite its therapeutic benefits, IR may inflict harm on healthy tissues, leading to significant complications [3,4], and can induce oxidative cellular injury, resulting in dysfunction of tissues and organs [5]. In females, the ovaries are extremely susceptible to radiation and are prone to damage during abdominal, pelvic, or total-body irradiation. IR accelerates depletion of primordial follicles, hence lowering ovarian reserve and adversely affecting ovarian function, resulting in early menopause and infertility in reproductive-aged women [6,7,8]. Further investigation and innovation are therefore essential to enhance the balance between effective tumor control and preservation of healthy tissues during radiotherapy [9]. Elucidating the molecular mechanisms underlying IR-induced ovarian injury is crucial for developing effective strategies to mitigate its adverse effects. When IR deposits energy into cells, it induces both direct and indirect cellular damage and promotes excessive generation of reactive oxygen species (ROS), which can damage nucleic acids, lipids, and proteins, triggering a variety of detrimental cellular consequences [10]. This cellular damage can cause multiple types of cell death, including apoptosis, necrosis, autophagic cell death, and ferroptosis, in both malignant and non-cancerous cells [10,11]. Among these mechanisms, ferroptosis has recently emerged as a potential contributor to IR-induced ovarian injury [12,13]. It is an iron-dependent form of cell death [14,15,16] that can be induced by IR through disturbances in iron homeostasis, increased lipid peroxidation, and impairment of the glutathione (GSH)–glutathione peroxidase 4 (GPX4) antioxidant defense system [17,18]. Notably, ferroptosis has been implicated in ovarian dysfunction, with iron accumulation, oxidative stress, and alterations in ferroptosis-related genes observed in experimental premature ovarian failure [19]. In addition, iron overload-induced ferroptosis in granulosa cells has been shown to impair oocyte maturation [20]. Given the essential role of granulosa cells in supporting oocyte survival, growth, and maturation, ferroptotic injury in these cells may indirectly compromise follicular function. These findings, together with the limited evidence from studies of IR-induced ovarian injury, suggest that ferroptosis may contribute to ovarian radiation toxicity.

However, ferroptosis is not the only cell death mechanism involved in IR-induced ovarian injury. The TAp63-PUMA-Noxa pathway is a well-known mediator of the activation of primordial follicle oocyte apoptosis caused by radiation-induced DNA damage [21,22]. Although inhibiting ferroptosis alone may not prevent TAp63-mediated oocyte apoptosis, it may help reduce granulosa cell injury and mitigate certain aspects of ovarian damage. The contribution of ferroptosis to IR-induced ovarian injury and the therapeutic potential of targeting this pathway remain incompletely understood. Thus, this review aims to critically evaluate current evidence on the role of ferroptosis in IR-induced ovarian injury, focusing on its underlying molecular mechanisms, interactions with other cell death pathways, and emerging ferroptosis-targeted therapeutic strategies, including their potential implications for mitigating ovarian damage.

## 2. Radiation-Induced Ovarian Toxicity

Ovaries are among the most susceptible organs to IR in females. Exposure to IR can substantially impair ovarian function and female fertility [6,23]. Therefore, elucidating the biological basis of ovarian radiosensitivity and the underlying cellular and molecular mechanisms is essential for understanding IR-induced ovarian injury.

### 2.1. Ovarian Structure and Radiosensitivity

The ovaries play a crucial role in female reproduction and endocrine regulation. The ovarian cortex is abundant in primordial and primary follicles embedded in a dense stroma, while the medulla comprises vascular, lymphatic, and neuronal plexuses [24]. Ovarian follicles are the primary functional units of the ovary, consisting of an oocyte surrounded by granulosa cells and an outer layer of theca cells [25]. The granulosa cells proliferate in the developing follicles and play an essential role in supporting oocyte maturation and follicle growth [26]. Therefore, the coordinated interaction of the oocyte, granulosa cells, and theca cells is critical for optimal ovarian function and female fertility. Primordial follicles are highly susceptible to IR [27], which can cause oocyte loss, granulosa cell injury, and stromal damage [28]. Mitotically active granulosa cells may die within a few hours of irradiation, while nearly 50% of immature oocytes may undergo structural damage following exposure to 2 Gy. Such damage can accelerate follicular depletion, reduce ovarian reserve, and lead to long-term impairment of ovarian function [23,29].

The degree of ovarian injury induced by radiation is dependent upon the radiation dosage, the volume of the ovary exposed, the fractionation routine, and the patient’s age during treatment [30]. Based on population models and clinical data, the half-lethal dose for human oocytes (LD50) is estimated to be below 2 Gy [31,32]. The effective sterilizing dose (ESD) is a fractionation equivalent dose that causes ovarian failure in 97.5% of individuals. The ESD decreases with age: 20.3 Gy in newborns, 18.4 Gy at 10 years, 16.5 Gy at twenty years, and 14.3 Gy at 30 years [30]. These characteristics contribute to the high risk of infertility and early menopause among female cancer survivors following radiotherapy, highlighting the importance of preserving ovarian function and fertility, particularly in young women exposed to pelvic irradiation [33,34].

### 2.2. Mechanisms of Radiation-Induced Ovarian Injury

**Figure 1:**
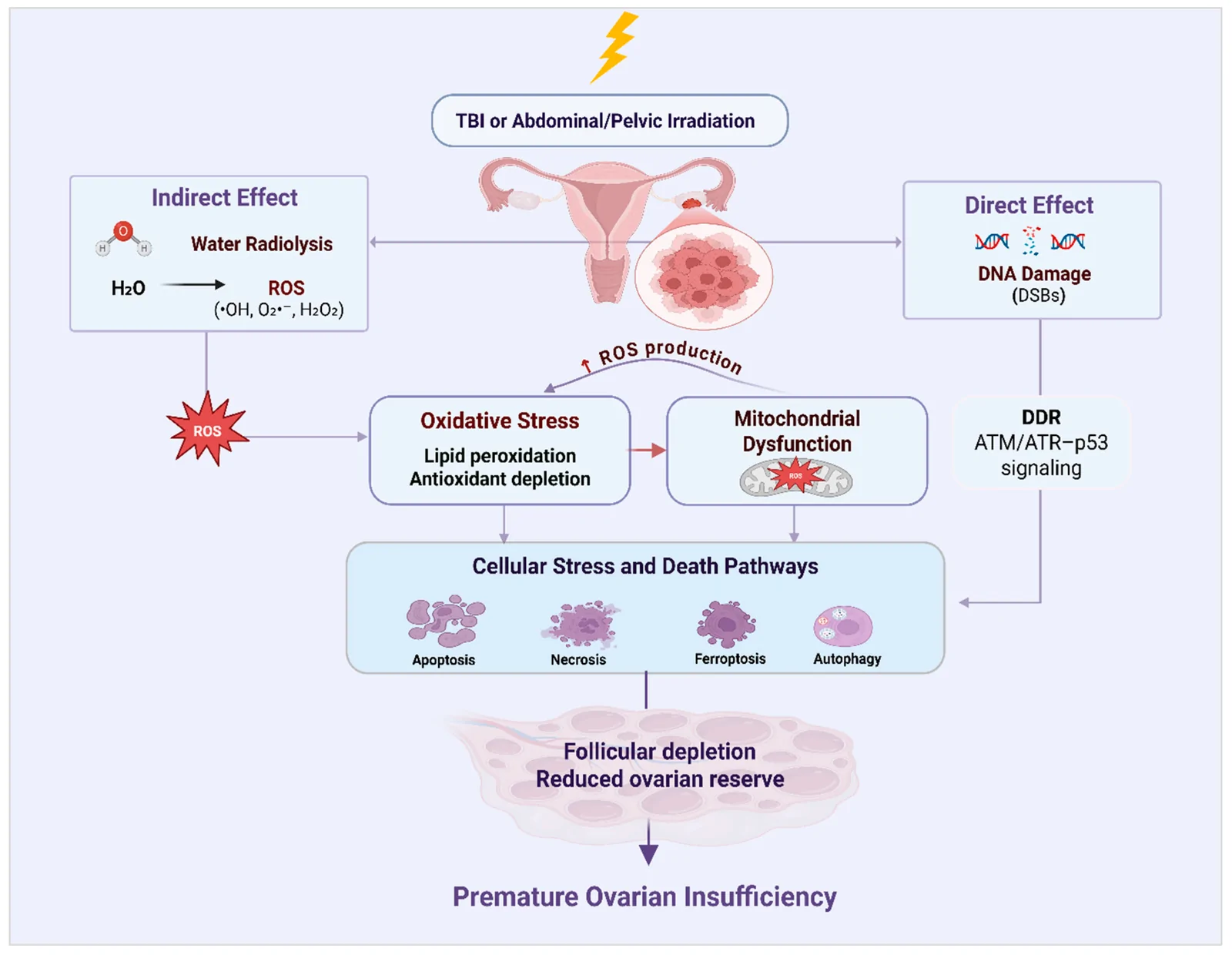
Overview of the mechanisms underlying radiation-induced ovarian injury. TBI, total body irradiation; ROS, reactive oxygen species; DSBs, double-strand breaks; DDR, DNA damage response; ATM, ataxia telangiectasia mutated; ATR, ataxia telangiectasia and Rad3-related. Created in BioRender. Khateeb, S. (2026) https://BioRender.com/8b00g8d.

IR induces ovarian injury via direct and indirect mechanisms, including direct DNA damage and ROS production, which can further damage cellular macromolecules [35,36]. These events promote oxidative stress, mitochondrial dysfunction, and cell death, ultimately contributing to follicular depletion and reduced ovarian reserve (Figure 1). Thus, understanding these molecular mechanisms is essential for elucidating IR-induced ovarian injury and developing effective strategies to preserve ovarian function during radiotherapy.

#### 2.2.1. Direct, Indirect Effects, and Oxidative Stress of IR

A direct effect occurs when radiation energy interacts directly with biological macromolecules, particularly DNA, resulting in base damage, single-strand breaks (SSBs), and double-strand breaks (DSBs), the latter being the most cytotoxic form of DNA injury [36,37]. Following DNA damage, the serine/threonine kinases ataxia telangiectasia mutated (ATM) and ataxia telangiectasia and Rad3-related (ATR) rapidly trigger the DNA damage response (DDR), activating downstream checkpoint kinases (CHK1/2) and p53 to induce cell-cycle arrest and assist in DNA repair [38]. These DNA-damage-response components act broadly across ovarian somatic and germ-cell compartments, but their downstream consequences differ by cell type: in granulosa, theca, and stromal cells, ATM/ATR–CHK1/2–p53 signaling primarily governs cell-cycle arrest, DNA repair, senescence, or apoptosis, whereas in dormant primordial oocytes, DNA damage primarily activates the CHK2–TAp63 apoptotic pathway described below. Cellular fate largely depends on the extent of DNA damage. Successful repair allows cells to resume proliferation, whereas excessive or irreparable DNA damage results in genomic instability and activates regulated cell death pathways or permanent cell-cycle arrest [39]. Although DNA is considered the primary target of direct radiation injury, proteins and membrane lipids may also undergo direct ionization, contributing to additional molecular and cellular dysfunction [37].

Conversely, the indirect effect arises from the chemical reaction of IR with cellular constituents, predominantly water [40], resulting in radiolysis and the production of ROS, such as superoxide anions (O_2_•^−^), hydroxyl radicals (•OH), and hydrogen peroxide (H_2_O_2_) [41,42]. These radiation-induced ROS can damage cellular macromolecules, thereby potentiating molecular and cellular injury [42,43]. The excessive ROS accumulation can surpass endogenous antioxidant defenses and disturb redox homeostasis, which results in oxidative stress [5]. IR disrupts mitochondrial functionality and increases intracellular oxidative stress, leading to an overproduction of ROS that damage the mitochondrial membrane and trigger apoptosis [44,45,46].

#### 2.2.2. Radiation-Induced Cell Death Pathways

Cellular death is an essential biological mechanism that maintains tissue homeostasis in normal physiological states; however, it can also be triggered by cellular stress and injury, particularly that resulting from IR. Post-irradiation, increased oxidative stress and permanent cellular injury initiate several cell death pathways that result in ovarian injury. Traditionally, cell death has been divided into apoptosis, autophagy, or necrosis [47]. Recent studies suggest a role for ferroptosis in radiation-induced ovarian injury [12,13].

Apoptosis is a regulated form of cell death characterized by distinct morphological changes, including cell shrinkage, nuclear condensation, and membrane blebbing [47]. When ROS-induced DNA damage cannot be repaired, p53 is activated, promoting the expression of pro-apoptotic genes while downregulating antioxidant defenses [48,49]. ROS-induced mitochondrial damage can cause mitochondrial dysfunction. Cytochrome c release subsequently activates caspases and promotes apoptosis, while Bcl-2 suppresses this process by blocking cytochrome c translocation from mitochondria [50]. In the oocyte specifically, DNA damage-induced apoptosis proceeds primarily through the TAp63-centered pathway; low-dose X-ray exposure has been associated with alterations in apoptosis-related markers in human ovaries, particularly increased NOXA and Bax expression in an age-dependent manner [22].

Additionally, dormant oocytes respond to DSBs primarily through the CHK2–TAp63 axis, in which sequential phosphorylation by CHK2 and CK1 activates TAp63 and triggers apoptosis [51,52]. Following TAp63 activation, the pro-apoptotic BH3-only proteins PUMA (p53-upregulated modulator of apoptosis) and Noxa are induced, contributing to DNA damage-induced apoptosis in primordial follicle oocytes and subsequent loss of ovarian reserve. The TAp63–PUMA–Noxa axis is a well-established mediator of DNA damage-induced primordial follicle oocyte apoptosis following irradiation. Kerr et al. [21] demonstrated that DNA damage-induced primordial follicle oocyte apoptosis and loss of fertility require TAp63-mediated induction of Puma and Noxa. Irradiated female mice lacking Chk2 exhibit preserved primordial follicles and fertility; similar effects are observed with combined p53/TAp63 deficiency, underscoring the importance of this axis in oocyte culling [53].

Necrosis is cell death resulting from severe damage to cells [54]. It is characterized by disruption of the integrity of the plasma membrane, leading to leakage of cytoplasmic contents into the extracellular space [55]. Unlike apoptosis, this release can cause inflammatory responses and harm to adjacent tissues [56]. On the other hand, autophagy is a process that entails the sequestration of cytoplasmic additives into autophagosomes and their subsequent lysosomal degradation [57]. Autophagy is involved in cell homeostasis and the disposal of harmful cellular components [58]. However, under some conditions such as oxidative stress or exposure to cytotoxic substances, autophagy can be dysregulated and contribute to cell death [59]. Both autophagy and apoptosis are involved in primordial follicle development and follicular atresia [60].

These cellular and molecular mechanisms are reflected in experimental models of IR-induced ovarian injury, which demonstrate alterations in ovarian morphology, follicular reserve, endocrine function, oxidative and inflammatory responses, cell death, and signaling pathways. Table 1 summarizes selected experimental studies that demonstrate these effects and associated mechanisms. Despite the substantial variability in radiation type, dose, irradiation field, and experimental model in the presented studies, they show a generally consistent pattern of IR-induced ovarian injury; one of the most prominent is follicular depletion. Studies that assessed ovarian follicle numbers indicated that irradiation causes a reduction in primordial follicles, with numerous studies also documenting a loss of developing follicles, an increase in atretic follicles, and histopathological alterations [12,13,61,62,63,64,65]. These findings suggest that IR influences several degrees of follicular boom, with primordial follicle depletion being a continual signal of ovarian reserve decline.

Structural ovarian damage was frequently accompanied by endocrine dysfunction. Across different experimental models, irradiation disrupted ovarian hormone homeostasis, with modifications most often regarding anti-Müllerian hormone (AMH), estradiol (E2), follicle-stimulating hormone (FSH), and luteinizing hormone (LH) [12,13,61,62,63,64,66,67]. AMH and E2 levels had been reduced in all investigations wherein those hormones have been evaluated. Conversely, FSH responses exhibited variability among experimental models, with increased ranges mentioned by He et al. [66], Zhang et al. [61], and Demyashkin et al. [63], whereas decreased levels were observed by Khateeb et al. [64] and Zhao et al. [13]; LH levels increased in He et al. [66] and Zhang et al. [61] but were reduced in Khateeb et al. [64]. Across experimental studies, ovarian irradiation has been associated with oxidative stress, inflammation, and apoptosis, as reflected by oxidative damage and altered antioxidant defenses, increased inflammatory mediators and related signaling, and changes in apoptosis-related markers [12,13,61,62,63,64,65,66,67,68,69,70].

Ovarian irradiation has also been associated with changes in cell proliferation, DNA damage and repair, and intracellular signaling pathways. Reduced cell proliferation was indicated by decreased Ki67 and/or PCNA expression [61,62,63,65], while increased γ-H2AX and 8-OHdG, DNA fragmentation, and alterations in ATM and RAD51 provided evidence of radiation-induced DNA damage and impaired repair [61,70]. Changes in PI3K/AKT, ERK/p38, and Nrf2-related signaling were also reported [13,61,62,63,64,66,70]. However, the direction of these molecular changes varied among studies. Nrf2 expression was decreased in Zhang et al. [61] and Khateeb et al. [64] but increased following irradiation in Xin et al. [62] and Zhao et al. [13]. Similarly, PI3K and AKT had been decreased in He et al. [66] and Demyashkin et al. [63], whereas increased PI3K and AKT were reported with the aid of Khateeb et al. [64], highlighting variability in radiation-induced molecular responses across experimental approaches. Recent studies have also suggested a possible role for ferroptosis in radiation-induced ovarian injury [12,13]. Although evidence is still limited, these results suggest that ferroptosis could be an additional mechanism contributing to ovarian impairment following irradiation.

**Table 1:** Experimental evidence of IR-induced ovarian injury.

Experimental Model/ Radiation Protocol	Cell Type/ Tissue Examined	Main Ovarian Outcomes	Associated Mechanisms/Pathways	Reference
Female Wistar rats/ Single-dose 5 Gy	Whole ovarian tissue	Histopathological alterations; Altered follicle counts	Follicular apoptosis	[68]
Female Wistar rats/ Single irradiation at 3.2, 4.0, or 4.8 Gy	Whole ovarian tissue	↓ preantral and antral follicles; ↑ atretic follicles; ↓ E2; ↑ FSH and LH; ↑IL-1β, IL-6, TNF-α and ROS; ↓PI3K and AKT; ↑ FOXO3a	Oxidative stress; inflammation; PI3K/AKT/FOXO3a signaling	[66]
Female C57BL/6 mice/ Single 5 Gy localized ovarian X-ray irradiation (1.5 Gy/min)	Whole ovarian tissue	↓ primordial–antral follicles; ↑ atretic follicles; ↑ FSH and LH; ↓ E2, and AMH; ↓ SOD and GSH-Px; ↑ MDA and ROS; ↓ Nrf2 and HO-1; ↑ TNF-α, IL-1β, IL-6, and cleaved caspase-3; ↓ Ki67 and PCNA; ↓ ATM and RAD51;	Oxidative stress; inflammation; apoptosis; impaired cell proliferation; impaired DNA repair; Nrf2/HO-1 signaling	[61]
Female C57BL/6 mice/ Single 3.2 Gy whole-body irradiation	Whole ovarian tissue	↓ primordial and preantral follicles; ↑ atretic follicles; ↓ E2 and AMH; ↑ MDA, Nrf2, and HO-1 ↑ Pho-NF-κB p65, NLRP3, caspase-1, ↑IL-1β, and IL-18; ↓ PCNA; ↑ Pho-P53, Bax, Cyto C, C-caspase-3, and C-PARP; ↓ Bcl-2;	Oxidative stress; inflammation; Nrf2/HO-1 signaling; NF-κB/NLRP3 signaling; impaired proliferation; apoptosis	[62]
Female Sprague–Dawley rats/ Single 3.2 Gy γ-irradiation	Whole ovarian tissue	Ovarian histopathological injury and follicular damage; ↓ E2 and AMH; oxidative ovarian damage; ↑ TLR-4, NF-κB, and TNF-α; ↑ p53, Bax, and caspase-3; ↓ Bcl-2	Oxidative stress; inflammation; TLR-4/NF-κB signaling; apoptosis	[67]
Female rats/ Single 2 Gy pelvic X-ray irradiation	Whole ovarian tissue	Ovarian histopathological damage; altered MDA levels ↑ apoptotic staining; ↑ necrotic staining;	Oxidative stress; apoptosis; necrosis	[69]
Female Wistar rats/20 Gy fractional local electron irradiation	Whole ovarian tissue	↓ primordial, primary, secondary, and tertiary follicles; ↑ atretic follicles; ↑ FSH; ↓ AMH; ↑ NOX4; ↑ IL-1β, IL-6, IL-4, and IL-10; ↓ Ki-67; ↑ caspase-3; ↓ PIK3R1, AKT1/2, and ERK1/2; ↑ p38	Oxidative stress; inflammation; apoptosis; impaired proliferation; suppression of PI3K/AKT and ERK signaling; p38 activation	[63]
Female Wistar albino rats/ Single-dose 6 Gy whole-body γ-radiation	Whole ovarian tissue	Ovarian histopathological injury; ↓ primordial and growing follicles; ↑ atretic follicles; ↓ AMH, estrogen, FSH, and LH; ↑ ROS and MDA; ↓ SOD and CAT; ↓ Nrf2; ↑ TNF-α and IL-1β; ↑ PI3K, AKT, and AMPK; ↑ TGF-β;	Oxidative stress; inflammation; PI3K/AKT/AMPK signaling	[64]
Female Wistar albino rats/ Single-dose 6 Gy whole-body γ-radiation	Whole ovarian tissue	Ovarian histopathological alterations ↓ TAC; ↑ 8-OHdG and DNA fragmentation; ↑ p53 and ERK1;	Oxidative stress; DNA damage; p53/ERK1 signaling	[70]
C57BL/6J mice/5 Gy lower-abdominal X-rays; ovarian explants/3 Gy; KGN cells/4.5 Gy X-rays	Mixed: whole ovarian tissue (in vivo/explant) and KGN granulosa-like cell line (in vitro)	↓ Primordial and primary follicles; ↑ atretic follicles; ↑ ROS and mitochondrial ROS; ↓ mitochondrial membrane potential; ↑ γ-H2AX; ↓ Ki67; ↑ TUNEL-positive cells and cleaved caspase-3	Oxidative stress; mitochondrial dysfunction; DNA damage; impaired proliferation; apoptosis	[65]
Female Sprague–Dawley rats/single 3.2 Gy whole-body γ-irradiation (0.46 Gy/min); primary ovarian granulosa cells/6 Gy γ-radiation	Mixed: whole ovarian tissue (in vivo) and primary granulosa cells (ex vivo)	↓ Primordial, preantral, and antral follicles; ↑ atretic follicles; ↓ E2 and AMH; ↑ Fe^2+^ and MDA; ↓ GSH and GPX4; mitochondrial alterations	Ferroptosis	[12]
Female Sprague–Dawley rats/single 2 Gy lower-abdominal irradiation with 6-MV X-rays	Whole ovarian tissue	Ovarian histopathological injury; ↓ primordial, primary, secondary, and antral follicles; ↓ FSH, E2, and AMH; ↑ ovarian iron, MDA, and ROS; ↓ GSH and SOD; ↓ GCLC and GPX4; ↑ TfR1; mitochondrial alterations	Ferroptosis	[13]

Note: ↑ indicates an increase; ↓ indicates a decrease.

## 3. Ferroptosis

Ferroptosis is a type of cell death that involves iron-mediated lipid peroxidation [71]. Ferroptosis is different from other forms of cell death in its biochemical and morphological properties [72]. Its biochemical properties include an increase in free iron, formation of ROS, and lipid peroxidation [18]. Its morphological features include mitochondrial shrinkage, high membrane density, lack of cristae, and disruption of the outer mitochondrial membrane [4]. In addition, several cellular mechanisms have been shown to affect ferroptosis susceptibility, including iron metabolism, the System Xc^−^–GSH–GPX4 pathway, and lipid metabolism [18]. Ferroptosis is also linked with the degeneration of ovarian follicles and primary ovarian insufficiency (POI), with experimental studies linking ferroptosis disruption to oocyte death and follicular atresia [73]. In parallel, previous studies have demonstrated that IR can induce ferroptosis [17,74]. These findings highlight the relevance of ferroptosis as a potential contributor to IR-induced ovarian injury.

### 3.1. Molecular Mechanisms of Ferroptosis

#### 3.1.1. Iron Metabolism and Iron Overload

Iron is a vital mineral that is necessary for many biological activities. Under physiological conditions, ferric iron (Fe^3+^) in complex with transferrin enters cells via transferrin receptor 1 (TfR1) and is subsequently incorporated into endosomes. In the endosome, Fe^3+^ is converted to ferrous iron (Fe^2+^) by the six-transmembrane epithelial antigen of the prostate 3 (STEAP3). Fe^2+^ then enters the cytosol through divalent metal transporter 1 (DMT1/SLC11A2), and the labile iron pool (LIP) is formed [75]. Iron in LIP is used by the mitochondria for their metabolism. The excess iron is stored in ferritin, which consists of ferritin heavy chain (FTH1) and ferritin light chain (FTL), or is excreted via ferroportin (SLC40A1/FPN) [18].

Disruption of iron metabolism can lead to intracellular labile iron accumulation, thereby promoting the Fenton reaction. FPN mediates cellular iron export and helps prevent ROS generation associated with intracellular iron overload. Reduced FPN expression has been reported in cells undergoing ferroptosis [76]. In addition, ferritin can undergo selective degradation through NCOA4-mediated ferritinophagy, releasing stored iron and expanding the LIP, thereby promoting the Fenton reaction [77]. In the Fenton reaction, Fe^2+^ reacts with H_2_O_2_ to generate Fe^3+^, •OH, and hydroxide ions (OH^−^). Fe^3+^ can subsequently be reduced back to Fe^2+^, sustaining ROS production [78]. The resulting ROS attack polyunsaturated fatty acids (PUFAs) within membrane phospholipids, initiating lipid peroxidation, membrane disruption, and ultimately ferroptotic cell death [79]. Therefore, disruption of iron homeostasis and expansion of the LIP represent key events promoting ferroptosis.

Clinical observations from systemic iron-overload disorders lend indirect support to the biological plausibility of iron-driven ovarian injury. Adolescent girls and women with transfusion-dependent β-thalassemia show reduced antral follicle counts, lower anti-Müllerian hormone, and diminished ovarian volume that correlate with serum ferritin and pituitary or ovarian iron burden [80,81], and iron-overloaded thalassemic mice display reduced AMH together with mitochondrial damage and fibrotic remodeling of ovarian tissue [80]. These associations are confounded by concurrent central hypogonadism resulting from pituitary iron deposition [80], so a causal, ferroptosis-specific contribution to follicular loss has not been identified in these populations. Nonetheless, the recurring pattern of iron-associated follicular, mitochondrial, and fibrotic ovarian injury in systemic iron-overload disorders is consistent with, and provides clinical context for, the ferroptosis-related mechanisms proposed here for IR-induced ovarian injury.

#### 3.1.2. Lipid Peroxidation

Lipids are the major structural component of cellular and organelle membranes. The membranes of mammals contain many phospholipids having at least one PUFA tail [82], which is crucial for membrane fluidity and supports membrane protein binding [83]. Among PUFA-containing phospholipids, phosphatidylethanolamines (PEs) containing arachidonic acid (AA; C20:4) or adrenic acid (AdA; C22:4) are considered important substrates for lipid peroxidation during ferroptosis [84]. Integration of these PUFAs into membrane phospholipids is mainly controlled by acyl-CoA synthetase long-chain family member 4 (ACSL4) and lysophosphatidylcholine acyltransferase 3 (LPCAT3), which play essential roles in PUFA metabolism and phospholipid remodeling [83]. ACSL4 converts free AA and AdA into acyl-CoA (AA/AdA-CoA). Then, LPCAT3 mediates the incorporation of AA/AdA-CoA into PE, thus forming PUFA-containing phospholipids, which are vulnerable to peroxidation [18]. Therefore, the ACSL4–LPCAT3 pathway enhances cellular susceptibility to ferroptosis, and depletion of ACSL4 or LPCAT3 may increase cellular resistance to ferroptosis [85,86]. The presence of bis-allylic hydrogen in the phospholipids containing PUFA makes them vulnerable to lipid peroxidation. Free radicals extract hydrogen atoms from these sites to produce lipid radicals that further react with molecular oxygen to generate lipid peroxyl radicals, which participate in lipid peroxidation chain reactions [78,87]. Furthermore, lipid peroxidation can also occur enzymatically through lipoxygenases (ALOXs), a family of enzymes that contain non-heme iron. ALOXs promote the peroxidation of AA/AdA-PE, producing cytotoxic AA/AdA-PE-OOH. These changes affect the fluidity and permeability of membranes and result in membrane dysfunction and potentially ferroptosis [88].

#### 3.1.3. System Xc^−^–GSH–GPX4 Pathway

**Figure 2:**
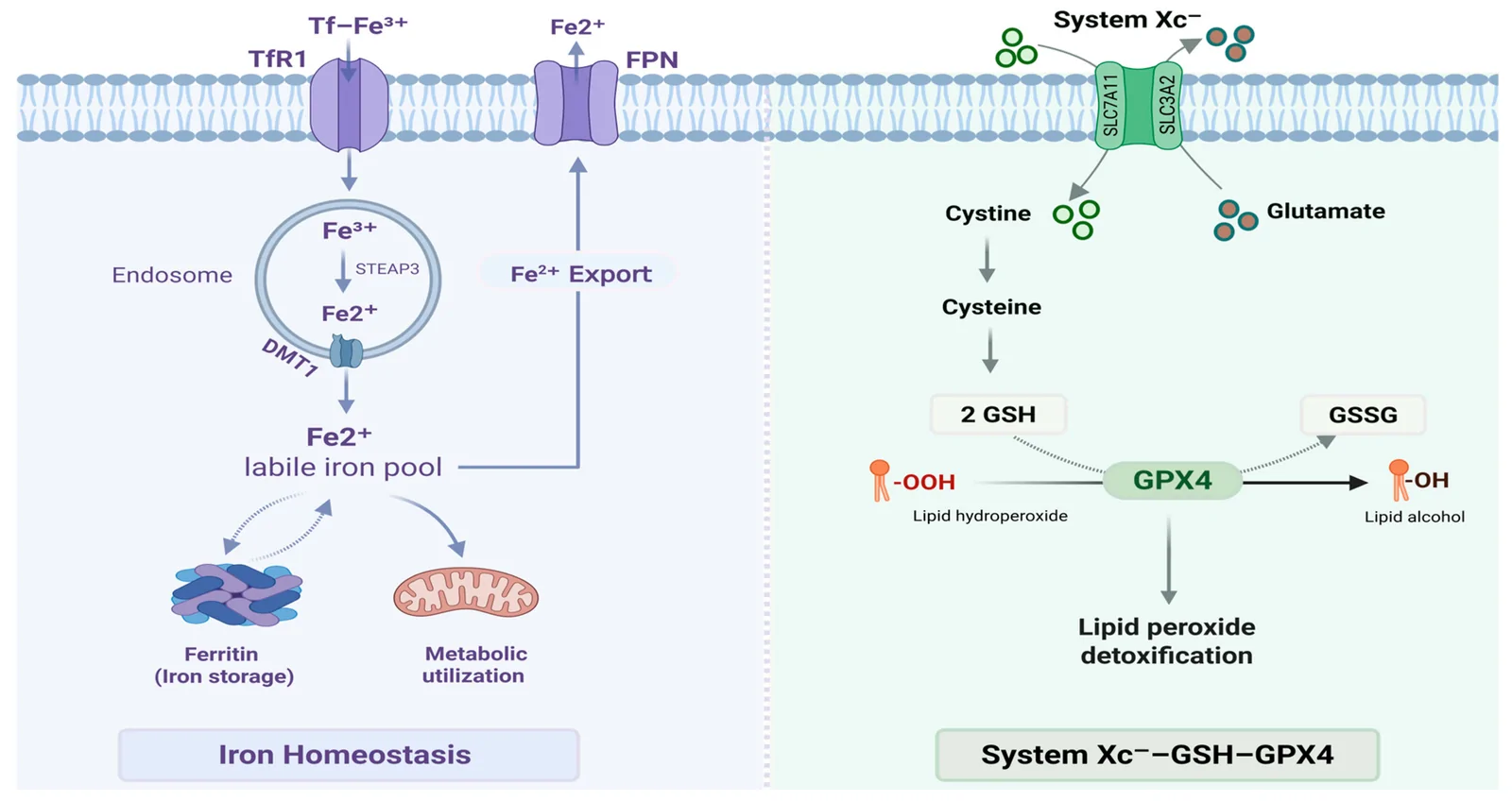
Physiological regulation of intracellular iron homeostasis and the System Xc^−^–GSH–GPX4 antioxidant defense pathway. DMT1, divalent metal transporter 1; Fe^2+^, ferrous iron; Fe^3+^, ferric iron; FPN, ferroportin; GPX4, glutathione peroxidase 4; GSH, reduced glutathione; GSSG, oxidized glutathione; LIP, labile iron pool; L-OH, lipid alcohol; L-OOH, lipid hydroperoxide; SLC3A2, solute carrier family 3 member 2; SLC7A11, solute carrier family 7 member 11; STEAP3, six-transmembrane epithelial antigen of the prostate 3; Tf, transferrin; TfR1, transferrin receptor 1. Created in BioRender. Khateeb, S. (2026) https://BioRender.com/slbq6wn.

The System Xc^−^–GSH–GPX4 pathway is an important cellular antioxidant mechanism that plays a central role in limiting lipid peroxide accumulation and maintaining redox homeostasis. System Xc^−^ is a membrane transporter comprised of the heavy chain SLC3A2 and the light chain SLC7A11. It imports extracellular cystine into cells by exchanging it for intracellular glutamate [89]. Following cellular uptake, cystine is reduced to cysteine, an essential substrate for GSH synthesis [90]. Thus, System Xc^−^ inhibition decreases cystine uptake and depletes intracellular GSH, allowing lipid peroxides to accumulate and promoting ferroptosis [91]. GSH is an essential intracellular antioxidant and a negative regulator of ferroptosis that serves as a crucial cofactor for GPX4 [92]. GPX4 is a selenium-dependent antioxidant enzyme that represents a crucial defense mechanism against lipid peroxidation [93,94]. GPX4 converts cytotoxic phospholipid hydroperoxides (L-OOH) to non-toxic phospholipid alcohols (L-OH) (Figure 2) and prevents the accumulation of lipid peroxides, thus maintaining the cellular redox homeostasis [95]. Consequently, reduced GPX4 expression promotes lipid peroxidation and increases cellular susceptibility to ferroptosis [96].

#### 3.1.4. Additional Regulatory Mechanisms of Ferroptosis

Besides the major mechanisms highlighted above, ferroptosis can be controlled by other regulatory processes. Among these regulators is Nrf2, which is an essential factor that controls cellular antioxidant defense and determines ferroptosis sensitivity through regulation of antioxidant defense and iron balance [97]. Under physiological conditions, Nrf2 is bound by Kelch-like ECH-associated protein 1 (Keap1), which leads to its ubiquitination and subsequent degradation by the proteasome. However, under oxidative stress conditions, the degradation of Nrf2 mediated by Keap1 is inhibited, thus allowing Nrf2 to accumulate and translocate to the nucleus. In the nucleus, Nrf2 will bind to antioxidant response elements (AREs), causing the transcription of antioxidant genes [98]. Nrf2 directly promotes the upregulation of SLC7A11, promoting cystine uptake, GSH synthesis, and GPX4 activity. It also influences ferritin (FTH1 and FTL) and ferroportin (SLC40A1), which are vital for iron storage and efflux [99,100]. Thus, activating Nrf2 reduces lipid peroxidation, inhibits ferroptosis, and enhances radioresistance. Conversely, the absence of Nrf2 disrupts those shielding mechanisms, resulting in iron accumulation, ROS generation, and improved vulnerability to ferroptosis [99,101,102].

P^53^ modulates cell cycle checkpoints, DNA repair mechanisms, and apoptosis as a critical tumor suppressor [103]. It promotes ferroptosis through the modulation of several downstream targets, including SLC7A11, spermine/spermidine N^1^-acetyltransferase 1 (SAT1), prostaglandin-endoperoxide synthase 2 (PTGS2), and glutaminase 2 (GLS2) [104]. Specifically, the suppression of SLC7A11 reduces cystine absorption, which in turn hinders GSH production and GPX4 activity, leading to increased ROS accumulation and promoting ferroptosis [105]. Additionally, p53 is involved in iron homeostasis by way of modulating transferrin and TFR1, resulting in increased intracellular iron levels and accelerated oxidative stress [106,107]. NADPH oxidases (NOXs) are important enzymes involved in redox signaling and are principal sources of cellular ROS. They use NADPH as an electron donor to generate O_2_•^−^ and H_2_O_2_. In response to cytokines and inflammatory signals, increased NOX activity can increase ROS generation and NADPH consumption, promoting oxidative damage and ferroptosis [108].

Ferroptosis is regulated by GPX4-independent antioxidant defense systems, particularly the FSP1–CoQ10–NAD(P)H and GCH1–BH4 pathways, which help suppress phospholipid peroxidation [109]. The FSP1 enzyme utilizes NAD(P)H to convert CoQ10 into ubiquinol (CoQH_2_), which then scavenges lipid peroxyl radicals and inhibits lipid peroxidation chain reactions [110]. This mechanism allows the FSP1–CoQ10–NAD(P)H pathway to restore cellular antioxidant capacity, inhibit ferroptosis independently of GPX4, and serve as a key complementary defense against ferroptotic cell death [109,111]. Similarly, the guanosine triphosphate cyclohydrolase 1 (GCH1)–tetrahydrobiopterin (BH4) pathway acts as another GPX4-independent defense system against ferroptosis. BH4 functions as a radical-trapping antioxidant and aids in the regeneration of CoQH_2_ and α-tocopherol, thereby reducing lipid peroxidation and ferroptosis [112,113].

### 3.2. Ferroptosis in IR-Induced Ovarian Injury

It should be noted at the outset that essentially all direct molecular evidence for ferroptosis in irradiated ovaries derives from the KGN granulosa-cell line, primary ovarian granulosa cells, or whole-ovary/whole-follicle homogenates that do not distinguish oocyte from somatic-cell contributions. To date, there is no direct experimental evidence that IR induces ferroptosis in oocytes themselves. Because granulosa cells directly support oocyte survival, growth, and maturation, granulosa-cell ferroptosis represents a plausible route by which follicles could be lost even in the absence of oocyte-intrinsic ferroptosis, but this indirect route, and any direct oocyte involvement, remain to be formally tested.

IR can induce ferroptosis in both malignant and normal cells [17]. In particular, IR promotes ferroptosis by disrupting iron homeostasis, affecting both iron transport [114] and storage, including the release of iron from ferritin [115]. IR can modify the expression of genes that regulate iron by enhancing iron uptake and diminishing iron storage and export [116], thereby increasing the intracellular LIP. The increase in redox-active iron subsequently enhances oxidative stress and lipid peroxidation, which in turn promotes ferroptosis. In addition to iron disturbances, the overproduction of ROS is another significant mechanism by which IR promotes ferroptosis. IR generates ROS through the radiolysis of cellular water, particularly •OH, which leads to oxidative damage of membrane lipids [17,117]. These ROS target PUFAs, producing fatty acid radicals (PUFA•). These radicals then interact with molecular oxygen to produce lipid peroxyl radicals (PUFA-OO•) and, subsequently, lipid hydroperoxides (PUFA-OOH). This chain reaction propagates lipid peroxidation in membrane phospholipids and ultimately promotes ferroptotic cell death [118].

**Figure 3:**
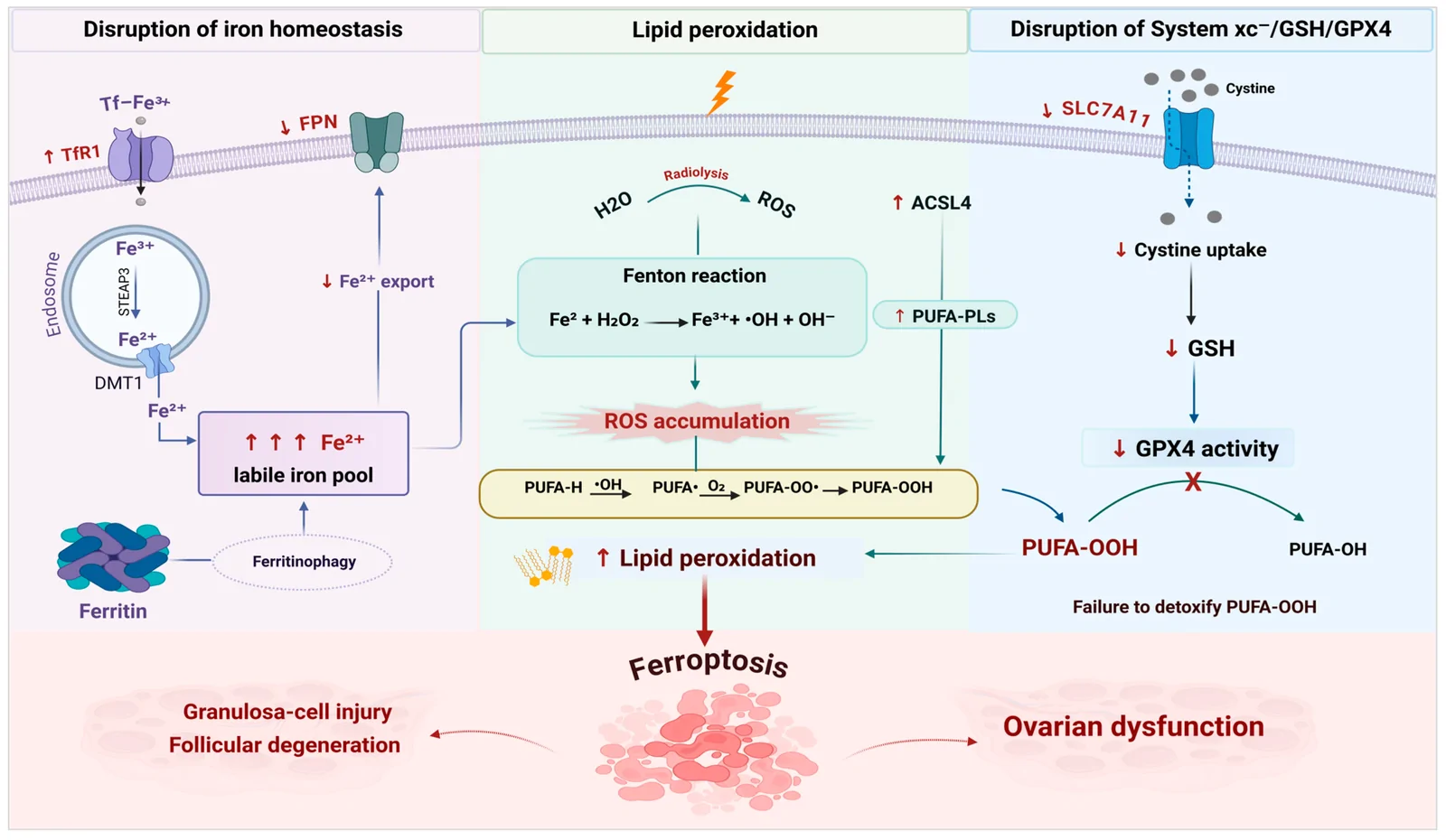
Mechanisms of ferroptosis in radiation-induced ovarian injury. ACSL4, acyl-CoA synthetase long-chain family member 4; DMT1, divalent metal transporter 1; FPN, ferroportin; GPX4, glutathione peroxidase 4; GSH, reduced glutathione; NCOA4, nuclear receptor coactivator 4; PUFA, polyunsaturated fatty acid; PUFA-PLs, PUFA-containing phospholipids; ROS, reactive oxygen species; SLC7A11, solute carrier family 7 member 11; STEAP3, six-transmembrane epithelial antigen of prostate 3; TfR1, transferrin receptor 1. Symbols: ↑ indicates an increase; ↓ indicates a decrease; the lightning bolt indicates ionizing radiation. Created in BioRender. Khateeb, S. (2026) https://BioRender.com/xozhjut.

IR further promotes ferroptosis by disrupting the System Xc^−^/GSH/GPX4 antioxidant defense axis. IR diminishes the expression of SLC7A11 [119,120], potentially through the activation of ATM kinase, which is known to suppress SLC7A11 expression and promote ferroptosis [121]. Similarly, GSH levels are reduced following exposure to IR [119], impairing the antioxidant defense mediated by GPX4 [94]. Furthermore, the expression of GPX4 has been shown to decrease after IR exposure [116,122,123], promoting ferroptotic cell death. In addition, IR can affect other ferroptosis-related genes, particularly ACSL4. Increased ACSL4 expression promotes the incorporation of PUFAs into membrane phospholipids, consequently enhancing their susceptibility to lipid peroxidation and ferroptosis [17,122,124]. Conversely, ACSL4 deficiency attenuates IR-induced ferroptosis and enhances radioresistance [17]. Ferroptosis has been implicated in various pathological conditions [16,125], and emerging evidence suggests a role in ovarian dysfunction [73]. Specifically, disruptions in iron homeostasis and the activation of ferroptosis may contribute to ovarian follicular degeneration [20,126]. These major mechanisms by which IR promotes ferroptosis are summarized in Figure 3.

As IR induces ferroptosis-related alterations, ferroptosis has been proposed as a potential mechanism for IR-induced ovarian injury. However, direct evidence supporting this association is currently limited to a small number of preclinical studies. The earliest evidence was reported by Zhao et al. [127], who showed that exposure of female Sprague–Dawley rats to 2 Gy X-rays affected ovarian function and altered the expression of many mitochondria-related genes, including notable down-regulation of GPX4; this study used bulk ovarian tissue rather than isolated follicular cell populations, so the cellular source of these changes cannot be assigned to a specific ovarian cell type. However, GPX4 reduction alone provides only suggestive evidence of ferroptosis involvement. More direct evidence was subsequently provided by Zhao et al. [123] using a 2 Gy X-ray-irradiated human ovarian granulosa (KGN) cell line. Irradiation promoted granulosa-cell death, accelerated intracellular iron accumulation, oxidative stress, and lipid peroxidation, with depletion of GSH, reduced GPX4 expression, elevated TfR1, and decreased FPN1. Also, mitochondrial morphological alterations were observed. These findings together provide compelling evidence of ferroptosis in IR-induced granulosa-cell damage.

Furthermore, Liu et al. [12] supplied additional in vivo and ex vivo proof by utilizing γ-irradiated female rats (3.2 Gy) and primary ovarian granulosa cells. The irradiation induced typical ferroptosis-related changes, including elevated Fe^2+^ and MDA levels, decreased GSH, reduced GPX4 expression, and mitochondrial changes. The results observed in both ovarian tissue and primary granulosa cells further support the role of ferroptosis in IR-induced ovarian damage. In line with these findings, Zhao et al. [13] presented more in vivo evidence in female Sprague–Dawley rats subjected to 2 Gy X-ray irradiation of the lower abdomen covering both ovaries. This irradiation increased levels of ovarian iron, MDA, ROS, and TfR1, while decreasing GSH and inhibiting GCLC and GPX4, along with mitochondrial changes. Collectively, these findings strengthen the evidence linking ferroptosis to IR-induced ovarian injury. This evidence base, however, remains limited to a single rat strain, the KGN cell line, and primary ovarian granulosa cells, with most studies using a 2 Gy dose. Moreover, the evidence is largely correlational rather than causal; the implications of these limitations for interpreting the review’s findings and for future research are addressed later in this review.

### 3.3. Ovarian Cell-Type Considerations in Ferroptosis Susceptibility

The mechanisms outlined in Section 3.1 have been characterized largely in non-ovarian cell systems, and their application to the ovary should account for the marked functional heterogeneity of its constituent cell types. Granulosa cells are metabolically active and mitotically engaged during follicular growth, with substantial mitochondrial activity and membrane turnover that could be expected to generate a correspondingly large pool of PUFA-containing phospholipid substrate for lipid peroxidation, consistent with granulosa cells being the ovarian cell type in which ferroptosis has so far been most directly documented above. In comparison, dormant primordial oocytes exhibit deep transcriptional quiescence while maintaining basal metabolic activity; their response to radiation is regulated by the CHK2–Tap63–PUMA/NOXA apoptotic pathway described above (Section 2.2.1). However, it has not been determined whether this quiescent state protects or makes cells more vulnerable to ferroptosis. Hence, the question of whether the effects of IR on oocytes are direct or indirect arises. At the moment, current data do not allow making this distinction. Only co-culture or specific genetic approaches will allow answering this question. Additionally, it is unknown whether primordial (dormant) and developing (antral or pre-antral) follicles differ in terms of susceptibility to ferroptosis. Primordial follicles usually represent the reserve of oocytes tied directly to fertility prospects, and because their behavior is different from that of growing follicles, they may possess different susceptibility profiles from those documented in growing follicles, which is a crucial issue. Finally, the local hormonal microenvironment may modulate this susceptibility. Estrogen has been reported in other tissues to limit ferroptosis by supporting antioxidant gene expression, and FSH drives the proliferative and metabolic state of granulosa cells that, as noted above, may itself influence ferroptosis susceptibility. Whether these endocrine signals modulate ferroptosis susceptibility specifically within irradiated ovarian follicles has not yet been tested and is noted here as a further avenue for investigation.

### 3.4. Crosstalk Between Ferroptosis and Other Cell Death Pathways

**Figure 4:**
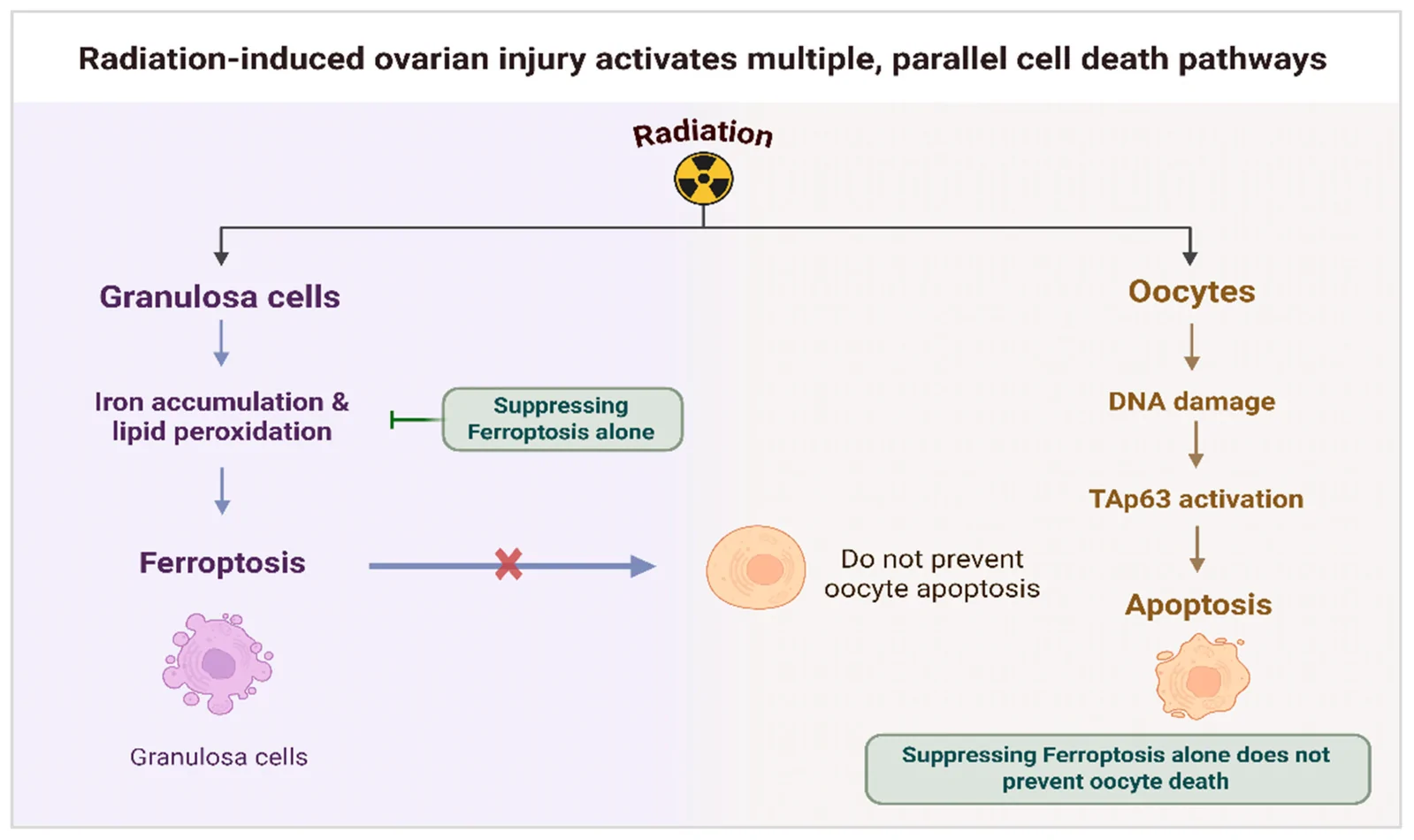
Radiation-related damage in the ovary triggers numerous pathways allowing cell types to die differently. In granulosa cells, radiation-induced accumulation of iron along with lipid peroxidation leads to ferroptosis. In oocytes, DNA damage following radiation exposure activates Tap63-mediated apoptosis. Created in BioRender. Khateeb, S. (2026) https://BioRender.com/0zl054i.

The pathways summarized in Figure 1 do not work independently, and their interactions illustrated schematically in Figure 4 are directly relevant to interpreting ferroptosis-targeted interventions. For instance, p53 demonstrates this interdependence by having a bi-directional nature. As a result, p53 can promote ferroptosis in some cases by transcriptionally repressing the expression of SLC7A11 and activating SAT1/GLS2, while in other instances, it rather inhibits ferroptosis due to CDKN1A/p21 expression and/or DPP4 activity inhibition. According to the cell types, the extent and duration of p53 activation, and the metabolic background, p53 may determine the advantages of one or another pathway. Specifically, in irradiated ovarian cells, p53 is located throughout the pro-apoptotic TAp63/PUMA/Noxa pathways in oocytes and the SLC7A11-dependent ferroptotic pathway described above; thus, it is necessary to understand how the mechanisms of cleavage between these two outputs occur in a specific irradiated ovarian cell.

Moreover, whether CHK2 also modulates ferroptosis in the ovary remains unknown. Given that ATM, an upstream kinase in the DNA damage response (DDR), can suppress SLC7A11 and promote ferroptosis, future studies should investigate whether CHK2 and related checkpoint kinases influence ferroptosis susceptibility in irradiated ovarian cells.

Autophagy can interact with ferroptosis because of NCOA4-associated ferritinophagy, described earlier. Ferritinophagy facilitates the release of iron, which can be consumed in the Fenton reaction, functioning as a rate-limiting factor in ferroptosis. Consequently, shifting autophagy activity in ovarian tissue implies a change in the sensitivity to ferroptosis, although the relationship has not been determined in cells that were exposed to radiation until now.

In the end, it is unknown how significant the contribution of ferroptosis and apoptosis to the occurrence of follicular atresia is and whether the two processes happen one after the other or simultaneously in the same follicle, which is relevant in terms of the conclusions of this review. If the inhibition of ferroptosis only drives the cells towards another death pathway instead of providing them with a possibility of survival, the positive effect of treatment regarding ovarian reserve may be much lower than it could be assumed from the results of ferroptosis-related studies. It is emphasized here that testing this concept–including the combination of ferroptosis inhibitors and apoptosis inhibitors in irradiated ovarian models in order to determine their protective capacity, whether additive, redundant, or causing some compensatory switch–will become one of the most important questions of further mechanistic research.

## 4. Targeting Ferroptosis

Emerging evidence linking IR to ferroptosis suggests the potential of targeting ferroptosis to reduce IR-induced tissue damage, including ovarian injury. Potential strategies for this approach include enhancing antioxidant defenses, maintaining iron homeostasis, and decreasing lipid peroxidation. It is important to note that the mechanistic trigger for ferroptosis differs between the injury models summarized in Table 2 and Table 3. In IR-induced ovarian injury, ferroptosis may be initiated through radiolysis-derived ROS and consequent lipid peroxidation acting on a background of DNA damage and DNA-damage-response signaling (Section 2.2.1 and Section 3.2). In contrast, in the non-radiation models of Table 3, ferroptosis is triggered by direct chemical or metabolic perturbation of iron or redox handling. These include cyclophosphamide- or cisplatin-derived reactive metabolites, direct cadmium–iron interactions, D-galactose-induced glycation and aging, or homocysteine-driven one-carbon metabolic stress. Because the upstream trigger differs, an intervention that restores GPX4 or limits iron accumulation in a chemically injured ovary is not guaranteed to be equally effective against a ROS/DNA-damage-driven insult, and its efficacy in the radiation setting should be regarded as a hypothesis generated by, rather than established by, the non-radiation data. Table 2 and Table 3 are therefore presented with distinct evidentiary weight throughout this section. Findings in Table 2 are treated as directly informative for IR-induced ovarian injury, whereas the mechanistically related but etiologically distinct findings in Table 3 are presented as supportive evidence for the general druggability of the GPX4, iron-homeostasis, and ACSL4-related pathways, and as a rationale for testing these same agents. Some of these agents, including ferrostatin-1 and deferoxamine, have already been tested in the irradiated setting, rather than as direct proof of efficacy against IR-induced ovarian ferroptosis.

One of the principal treatment strategies identified for inhibiting ferroptosis in ovarian injury is the maintenance of GPX4 activity. Direct evidence from ovarian injury models induced by IR suggests that modifying GPX4-dependent antioxidant defenses can alleviate ferroptosis triggered by IR (Table 2). Preliminary findings have shown that sphingosine-1-phosphate (S1P) can restore the GPX4 expression diminished by IR in rat ovaries [127]. Further studies have shown the protective effects of S1P in irradiated KGN cells through the restoration of GPX4 and the reduction in GSH depletion [123]. Additionally, a recent investigation utilizing Zuogui Wan demonstrated the restoration of both GPX4 and GSH while inhibiting ferroptosis via the miR-21-5p/DNMT3b axis [12]. In evaluation, S1P was found to preserve the Nrf2–GCLC–GSH–GPX4 pathway, leading to increased levels of GCLC, GSH, and GPX4 in irradiated rat ovaries [13]. In the KGN granulosa-cell line, S1P acted directly on irradiated granulosa cells in vitro; in the rat model reported by Zhao et al. [127] and Zhao et al. [13], S1P was administered systemically, and its effects were assessed in whole-ovarian tissue, so a granulosa-cell-autonomous mechanism cannot be distinguished from an indirect (e.g., vascular, stromal, or endocrine) effect in vivo.

Further evidence from non-radiation ovarian damage models supports GPX4 as a potential therapeutic target (Table 3). Despite variations in the experimental models and therapies utilized, several approaches focus on preserving GPX4-dependent antioxidant defense. Proanthocyanidins suppressed ferroptosis through the SIRT1/Nrf2/GPX4 pathway [128], while human umbilical cord mesenchymal stem cell-derived exosomes (hUMSC-Exos) enhanced Nrf2/GPX4 signaling [129] in cyclophosphamide-induced premature ovarian insufficiency (POI) mice and KGN cells. Similarly, 3D hUCMSC spheroids maintained GPX4 activity through Nrf2-mediated antioxidant regulation [130], while Oviductus Ranae suppressed ferroptosis by regulating the GPX4/ACSL4 axis [131]. Collectively, these findings provide additional support for investigating GPX4-dependent antioxidant defense as a potential therapeutic target for IR-induced ovarian injury.

Targeting lipid peroxidation represents another approach to suppress ovarian ferroptosis. Ferrostatin-1, a radical-trapping antioxidant, has demonstrated protective benefits in multiple non-radiation ovarian damage models (Table 3). Ferrostatin-1 has been shown to mitigate ferroptosis in several contexts: in granulosa cell injury induced by homocysteine [132], in a mouse model of POI due to Basonuclin 1 (BNC1) deficiency [73], in vitro granulosa cell injury caused by cis-dichlorodiammineplatinum(II), and in cases of premature ovarian failure in rats [133]. Additionally, it has been effective in protecting against ovarian damage induced by cadmium (Cd) [134] and in ovarian injury in the offspring of mice and ovarian granulosa cells (in vitro) exposed to maternal polystyrene nanoplastics (PS-NPs) [135]. Furthermore, ferrostatin-1 has recently been shown to attenuate IR-induced ovarian injury in vivo, providing direct evidence for the protective effects of ferroptosis inhibition [13]. These findings suggest that direct inhibition of ferroptotic lipid damage can protect ovarian cells and maintain ovarian function.

Another strategy to inhibit ferroptosis involves targeting iron accumulation. Deferoxamine, an FDA-approved iron chelator, binds to non-transferrin-bound free iron [136,137]. As a result, deferoxamine is recognized as a ferroptosis inhibitor and has been shown to prevent IR-induced ferroptosis in KGN cells following exposure to 2 Gy X-ray irradiation. Deferoxamine not only reduced iron accumulation but also increased FPN1 expression while decreasing TfR1 expression in irradiated cells [123]. In line with the therapeutic importance of iron modulation, Zuogui Wan was found to decrease ovarian iron levels in irradiated rats [12]. Additionally, S1P was observed to reduce both iron accumulation and TfR1 expression [13]. Supporting data from non-radiation ovarian damage models further emphasize the therapeutic target of iron homeostasis [130,131]. These findings underscore the potential of iron regulation as a strategy to mitigate ferroptosis in IR-induced ovarian injury.

PUFA metabolism represents another potential target for limiting ferroptosis. Evidence from non-radiation ovarian injury models indicates that various interventions, including ferrostatin-1, verteporfin, LW6, and Oviductus Ranae, reduce ACSL4 expression [73,131,132,133,134,135] (Table 3). These findings suggest that targeting ACSL4-associated lipid metabolism could mitigate ovarian ferroptosis. However, direct therapeutic targeting of ACSL4 has not yet been evaluated in IR-induced ovarian injury. Together, these findings highlight several potential strategies for limiting ovarian ferroptosis. Nevertheless, direct evidence in IR-induced ovarian injury remains limited, and further studies are needed. Before these strategies can be used clinically, their potential effects on tumor radiosensitivity must also be considered.

**Table 3:** Preclinical evidence for ferroptosis-targeted interventions in non-radiation ovarian injury models.

Therapeutic Agent	Experimental Model	Ferroptosis-Related Mechanism/ Main Findings	Reference
Ferrostatin-1	Homocysteine-induced KGN granulosa cell injury	Suppressed ferroptosis through TET1/2-mediated regulation of DNA methylation; reduced Fe^2+^, ROS, and MDA, increased GSH, GPX4, and SLC7A11, and decreased ACSL4 and DMT1	[132]
Verteporfin and Ferrostatin-1	BNC1 deficiency-induced POI mouse	Ferrostatin-1 partially reversed BNC1 deficiency-induced lipid peroxidation, restored GPX4 expression; Verteporfin suppressed NF2–YAP–TFRC/ACSL4-mediated ferroptosis, reducing TFRC, Fe^2+^, and lipid ROS	[73]
Ferrostatin-1	Cis-dichlorodiammineplatinum(II)-induced granulosa cell injury in vitro and premature ovarian failure in rats in vivo	Inhibition of ferroptosis through GPX4 as potential protective targets; reduced Fe^2+^, ROS, and MDA, restored GSH/SOD and mitochondrial function, and increased NRF2, HO-1, and GPX4 expression	[133]
hUMSC-Exos	Cyclophosphamide-induced POI mice and KGN cells	Suppressed ferroptosis via the Nrf2/GPX4 pathway; ↑ Nrf2, and GPX4, ↓ Fe^2+^, ROS, and lipid peroxidation	[129]
Ferrostatin-1	Cd-induced ovarian injury in ICR mice	Suppressed ferroptosis through the ACSL4/GPX4 axis; reduced Fe^2+^, 4-HNE, and MDA, increased GSH and GPX4, decreased ACSL4	[134]
3D hUCMSC spheroids	H_2_O_2_-induced ovarian GCs and POF rats	Suppressed ferroptosis via Nrf2 signaling; ↓ iron accumulation and lipid peroxidation, restored GSH metabolism, prevented GPX4 inactivation.	[130]
Oviductus Ranae	D-galactose-induced ovarian aging rats and ovarian GCs	Inhibited ferroptosis via the GPX4/ACSL4 axis; ↓ Fe accumulation and lipid peroxidation, ↑ GSH and GPX4, ↓ ACSL4, ↑ SLC7A11	[131]
Ferrostatin-1 and LW6	Maternal PS-NPs-induced ovarian injury in mice offspring (in vivo) and ovarian granulosa cells (in vitro)	Ferrostatin-1 and LW6 attenuated molecular markers of ferroptosis (ACSL4, FTH1, SLC7a11, and GPX4)	[135]
Proanthocyanidins	Rat model of ovarian ischemia-reperfusion injury (in vivo)	Inhibited GPX4-dependent ferroptosis through activation of the SIRT1/Nrf2/GPX4 pathway; restored ferroptosis-related proteins reduced oxidative stress	[128]

Note: ↑ indicates an increase; ↓ indicates a decrease.

### Balancing Ovarian Protection and Tumor Radiosensitivity

One of the critical aspects of translating any ferroptosis-based ovarian protection method is the fact that similar mechanisms of GPX4 preservation, iron chelation, and limitation of lipid peroxidation might protect tumor cells against IR-induced ferroptotic death. These findings suggest that susceptibility to ferroptosis may contribute to tumor radioresistance and radiosensitivity. Moreover, increased GPX4 levels have been associated with ferroptosis resistance in cancer cells, highlighting the potential risk that strategies protecting normal tissues may also protect tumor cells. Similar to that, a conflict between the usefulness of interventions is created: the methods where the window of protection is increased through ferroptosis inhibition are opposed by the methods focused on increasing the window of radiosensitivity through ferroptosis induction. None of the ovarian-protection studies summarized in Table 2 concurrently assessed tumor control or tumor radiosensitivity. Ovary-selective protection could in principle be pursued through spatial and temporal targeting rather than systemic ferroptosis inhibition: examples include localized or intra-ovarian/intra-arterial drug delivery, ovary-targeted nanoparticle carriers, and timing administration to the peri-irradiation window in the ovary while sparing systemic exposure during tumor-bed irradiation; these approaches remain to be tested for the agents discussed in this section. In terms of clinical positioning, a pharmacological ferroptosis-targeted approach would differ from the two currently used fertility-preservation strategies in complementary ways. The surgical nature of ovarian tissue cryopreservation and transplantation requires careful planning and is normally reserved for high-risk ovarian failure patients. There is no risk of tumor cell damage since the tissue is removed from the radiation site completely. GnRH agonist co-administration is a non-invasive procedure that can occur during radiation therapy. Although GnRH agonist efficacy in the prevention of radiation-induced ovarian failure is not well established, the effects of GnRH agonists are not related to gonadotropins. The pharmacological approach that employs the mechanism of ferroptosis via medication could provide a significant benefit since it delivers a therapy non-invasively without surgery. However, its therapeutic effects on the ovaries and on the tumor remain unconfirmed.

## 5. Limitations

When analyzing the findings presented in this review, some limitations should be taken into account. Specifically, the existing direct evidence of ferroptosis in IR-induced ovarian damage is limited to a single rat strain and a narrow range of cellular models, including KGN cells and primary ovarian granulosa cells. This evidence was gathered almost exclusively after a single-incident radiation exposure (particularly 2 Gy). This approach stands in stark contrast to the fractionated protocols that are utilized for pelvic radiotherapy as well as to differences between rodents and large mammals (including humans) in the processes of folliculogenesis, follicle size, and hormone response [138,139]. Also noteworthy is that much of the mechanistic evidence that was accumulated is of a correlative nature, whereby there is an increase in ferroptosis markers with irradiation, and its further reversal by pharmacological agents, but no definitive proof was provided in the ovarian tissue using genetic methods. Recent international consensus guidelines for the ferroptosis field recommend combining genetic and pharmacological approaches to establish a causal role, since chemical inhibitors and inducers can have off-target effects [140]. In addition, most of the endpoints summarized in this review were measured in whole-ovarian tissue or serum rather than in isolated cell populations, limiting cell-type resolution, and much of the therapeutic evidence discussed derives from non-radiation ovarian injury models with a distinct upstream trigger. Finally, none of the ovarian-protection studies reviewed here concurrently assessed tumor radiosensitivity, so the net effect of these strategies on the therapeutic ratio of pelvic radiotherapy remains unknown.

## 6. Future Insights and Research Directions

Building on the limitations discussed in the preceding section, the following priorities emerge for future research:

- Establish causality using conditional, ovarian-cell-type-specific genetic models (e.g., granulosa-cell Gpx4 or Acsl4 deletion) in irradiated animals, in line with current consensus recommendations for ferroptosis validation [138].
- Extend experimental models beyond a single rat strain and the KGN cell line to additional rodent strains, primary human granulosa cells, and large-animal or human ovarian tissue, given the documented differences in follicular dynamics between species [136,137].
- Test clinically relevant fractionated radiation regimens alongside acute single-dose exposures across the dose range relevant to age-dependent ovarian failure.
- Apply single-cell or cell-type-resolved approaches to determine whether ferroptosis occurs directly in oocytes or is confined to granulosa cells and other somatic populations.
- Directly compare and combine ferroptosis inhibition with apoptosis-targeted strategies, such as those addressing the Tap63–PUMA–Noxa axis, to test whether dual targeting offers superior protection of ovarian reserve.
- Incorporate concurrent tumor-control and tumor-radiosensitivity endpoints into ovarian-protection studies, and explore ovary-selective delivery strategies to minimize any risk of tumor protection.
- Develop standardized, clinically translatable biomarkers of ovarian ferroptosis to support future human trials of ovarian-protective agents.

## 7. Conclusions

Ferroptosis has emerged as a potential contributor to IR-induced ovarian injury. Current evidence suggests that IR promotes ferroptosis by disrupting iron homeostasis, increasing lipid peroxidation, and impairing the System Xc^−^–GSH–GPX4 antioxidant defense axis. On the other hand, radiation also activates the well-established Tap63–PUMA/NOXA apoptotic pathway in oocytes, highlighting the involvement of multiple cell death mechanisms in ovarian injury. Nevertheless, targeting ferroptosis may help reduce granulosa cell injury and mitigate certain aspects of ovarian damage. Existing strategies primarily focus on preserving GPX4-dependent antioxidant defense, limiting iron accumulation and lipid peroxidation, and regulating PUFA-related lipid metabolism. However, direct therapeutic evidence in IR-induced ovarian injury remains limited, and much of the supporting evidence comes from other ovarian injury models. Further studies must be carried out to confirm these findings and establish causal relationships. In particular, a critical gap in the existing literature involves the need to assess the utility of ferroptosis-targeting interventions in ovarian protection because, thus far, scientists have not established whether the mechanisms of GPX4 protection, iron removal, and restriction of lipid peroxidation play any role in the preservation of tumor radiosensitivity; this means that the same processes could potentially help prevent tumor cells from ferroptosis induced by radiotherapy. Resolving this question, ideally through ovary-selective delivery strategies that avoid systemic ferroptosis inhibition, represents a prerequisite for clinical translation rather than a secondary caveat.

## Figures and Tables

**Table 2:** Preclinical studies of ferroptosis-related interventions in IR-induced ovarian injury.

Therapeutic Agent	Experimental Model	Ferroptosis-Related Mechanism/Main Findings	Reference
Sphingosine-1-phosphate	Female Sprague–Dawley rats exposed to a single 2 Gy dose of 6-MV X-rays directed to the lower abdominal reproductive area	Ferroptosis was not directly investigated; sphingosine-1-phosphate restored radiation-suppressed GPX4, UQCRH, and MICU2 expression	[127]
Sphingosine-1-phosphate	KGN cells exposed to 2 Gy X-ray irradiation	Inhibited radiation-induced ferroptosis by upregulating GPX4; Restored GPX4 expression, reduced intracellular Fe^2+^ accumulation, increased GSH, decreased MDA, restored SOD/CAT/GSH-Px activities, increased FPN1 and decreased TfR1	[123]
Deferoxamine	KGN cells exposed to 2 Gy X-ray irradiation	Iron chelation and inhibition of iron-dependent ferroptosis; Deferoxamine reduced intracellular Fe^2+^ and MDA levels, restored GSH and GPX4 expression, increased FPN1 and decreased TfR1 expression, enhanced SOD, CAT, and GSH-PX activities	[123]
Zuogui Wan	Female Sprague–Dawley rats exposed to 3.2 Gy whole-body γ-irradiation; primary ovarian granulosa cells exposed to γ-irradiation	miR-21-5p/DNMT3b axis; ZGW inhibited radiation-induced ↑ Fe^2+^ and MDA, restored GSH, and GPX4 levels, increased miR-21-5p expression, which targeted DNMT3b and suppressed its expression	[12]
Sphingosine-1-phosphate	Female Sprague-Dawley rats received a single 2 Gy dose of 6-MV X-rays directed to the lower abdominal region encompassing both ovaries.	Suppressing ferroptosis via activating the Nrf2-GCLC-GPX4 axis; ↑ GCLC and GPX4, ↓ TfR1; ↓ iron accumulation, ROS, and lipid peroxidation, ↑ GSH and SOD, preservation of mitochondrial membrane potential	[13]

Note: ↑ indicates an increase; ↓ indicates a decrease.

## Data Availability

No new data were created or analyzed in this study. Data sharing is not applicable to this article.
